# Low Levels of Mouse γδ T Cell Development Persist in the Presence of Null Mutants of the LAT Adaptor

**DOI:** 10.3390/ijms262412186

**Published:** 2025-12-18

**Authors:** Mikel M. Arbulo-Echevarria, Luis M. Fernandez-Aguilar, Elke Kurz, Inmaculada Vico-Barranco, Raquel Muñoz-Fernández, Isaac Narbona-Sánchez, Manuel Carrasco, Bernard Malissen, Michael L. Dustin, Enrique Aguado

**Affiliations:** 1 Department of Biomedicine, Biotechnology and Public Health, Universidad de Cádiz, 11002 Cádiz, Spain; mmartinezdeae@outlook.com (M.M.A.-E.); luismi.fernandez@gm.uca.es (L.M.F.-A.); inmaculada.vico-barranco@jc-discovery.com (I.V.-B.); raquel.mfernandez@uca.es (R.M.-F.); isaac.narbona@uca.es (I.N.-S.); manuel.carrasco@uca.es (M.C.); 2 Biomedical Research and Innovation Institute of Cádiz (INiBICA), 11009 Cádiz, Spain; 3 Kennedy Institute of Rheumatology, Nuffield Department of Orthopaedics, Rheumatology and Musculoskeletal Sciences, University of Oxford, Oxford OX1 7FY, UK; elke.kurz@kennedy.ox.ac.uk (E.K.); michael.dustin@kennedy.ox.ac.uk (M.L.D.); 4 Centre d’Immunologie de Marseille-Luminy (CIML), Aix Marseille Université, Institut National de la Santé et de la Recherche Médicale (INSERM), Centre National de la Recherche Scientifique (CNRS), 13009 Marseille, France; bernardm@ciml.univ-mrs.fr; 5 School of Medical Technology, Henan Medical University, Xinxiang 453003, China; 6 Centre d’Immunophénomique (CIPHE), Aix Marseille Université, Institut National de la Santé et de la Recherche Médicale (INSERM), Centre National de la Recherche Scientifique (CNRS), 13009 Marseille, France; 7 Chinese Academy of Medical Science Oxford Institute, Nuffield Department of Medicine, University of Oxford, Oxford OX3 7BN, UK

**Keywords:** LAT, TCR, signaling, γδ T cell

## Abstract

Activation through the T cell receptor (TCR) initiates a signaling cascade in T cells that induces extensive molecular and cellular changes. The adaptor protein Linker for Activation of T cells (LAT) plays an essential role in transducing activation and regulatory signals downstream of the TCR. Phosphorylation of LAT tyrosine residues recruits multiple signaling proteins, leading to the assembly of the LAT signalosome, which is crucial for relaying signals that regulate T cell development and function. We previously showed that substitution of a negatively charged amino acid segment preceding the fifth tyrosine residue of LAT (Tyr127 in humans or Tyr132 in mouse LAT) enhances some early TCR signaling events, whereas downstream responses, such as Ca^2+^ influx and Erk phosphorylation, are partially inhibited. To investigate the physiological relevance of this segment in vivo, we generated a new LAT knock-in mouse strain (*Lat*^NIL^) in which the negatively charged segment was replaced with a non-charged sequence. Unexpectedly, this mutation led to an alternative splicing event in the *Lat* gene that excluded exons 6 and 7, resulting in a frameshift, a premature stop codon at residue 145, and the loss of the six C-terminal tyrosine residues of LAT. Homozygous *Lat*^NIL/NIL^ mice showed a phenotype similar to that of LAT-knockout and *Lat*^4YF^ mice (in which the four C-terminal tyrosines had been mutated to phenylalanine). Interestingly, homozygous *Lat*^NIL/NIL^ mice exhibited a distinct population of γδ T cells in lymphoid organs, which has not been observed in LAT-KO or *Lat*^4YF^ mice. These γδ T cells expressed higher levels of CD27 compared to those in wild-type and LAT-KO mice, suggesting altered activation or differentiation states. Together, these data highlight how subtle alterations in LAT structure can profoundly impact T cell signaling and lineage composition.

## 1. Introduction

T cell-mediated immunity plays a crucial role in the host defense against pathogens and cancer. Its capacity for antigen-specific recognition enables the generation of intense yet tightly regulated responses against countless potential threats. Following specific binding of the T cell receptor (TCR), a cascade of finely controlled intracellular signaling events is triggered, leading to T cell activation, proliferation, and differentiation. This signaling network also operates during T cell development, where it is essential for the generation of cells capable of mounting protective immune responses while preventing the emergence of autoreactive cells.

At the core of these signaling pathways lies the Linker for Activation of T cells (LAT), an essential adaptor protein that coordinates downstream signaling events following TCR engagement [1,2,3,4]. LAT functions as a molecular scaffold that recruits and organizes multiple signaling molecules into a multiprotein complex known as the LAT signalosome [5,6]. This complex is crucial for translating TCR engagement into intracellular signals that drive T cell development, activation, and differentiation. LAT activity primarily depends on four conserved C-terminal tyrosine residues located at positions 136, 175, 195, and 235 (mouse numbering). Phosphorylation of these residues enables the binding of Grb2, phospholipase C-γ1 (PLC-γ1) and the Gads-SLP-76 complex [7,8]. The critical role of LAT in T cell development was demonstrated through the generation of LAT-deficient (LAT-KO) mice [9,10], which exhibited a complete absence of peripheral T cells due to a developmental block at the CD4^−^CD8^−^ double-negative (DN) stage. The in vivo significance of the four C-terminal tyrosine residues of LAT was further examined in a knock-in strain harboring Tyr-to-Phe substitutions at positions 136, 175, 195, and 235 (*Lat*^4YF^) [10,11]. Homozygous *Lat*^4YF/4YF^ mice displayed a phenotype identical to that of LAT-KO mice, characterized by a complete lack of peripheral T lymphocytes due to developmental arrest at the DN stage, while B and NK cell development remained unaffected. Collectively, these findings demonstrate that phosphorylation of the four C-terminal tyrosine residues of LAT is indispensable for pre-TCR signaling and T cell development, underscoring the essential role of LAT signalosome assembly during β-selection at the DN stage.

These results established LAT as a pivotal mediator of activation signals downstream of the TCR and pre-TCR. However, analysis of a knock-in mouse strain expressing a Tyr-to-Phe mutation at position 136 (*Lat*^Y136F^) revealed an unexpected regulatory function for LAT [12]. While in Jurkat cells this mutation abrogated the LAT-PLC-γ1 interaction and Ca^2+^ influx [7,8,13], homozygous *Lat*^Y136F/Y136F^ mice displayed peripheral accumulation of polyclonal helper T (TH) cells that chronically produced large amounts of type 2 cytokines. This exaggerated TH2 differentiation led to tissue eosinophilia and massive maturation of plasma cells secreting IgE and IgG1 immunoglobulins, revealing for the first time a critical role of LAT in maintaining T cell homeostasis [12,14].

To explore non-tyrosine-based motifs that might contribute to the regulatory functions of LAT, several studies of early TCR signaling events have identified a transient interaction between the open active form of Lck and LAT, which appears to downregulate Lck kinase activity [15,16]. This interaction is thought to be mediated by a negatively charged region spanning amino acids 112–126 of human LAT, possibly facilitated and fine-tuned by weakly polar interactions with tyrosine, including C-H...π and π...π interactions [17]. In a previous work using J.CaM2.5 cells (a LAT-deficient Jurkat derivative) we demonstrated that replacing this region with an uncharged amino acid segment produced dual effects on TCR signaling: enhanced proximal signaling events but reduced downstream responses, including Ca^2+^ influx and Erk phosphorylation [18]. Furthermore, phosphorylation of LAT has been shown to be regulated through the formation of an Lck-ZAP70-LAT complex [19]. Collectively, these findings underscore the finely tuned balance that governs TCR signal transduction.

To investigate the physiological relevance of this negatively charged LAT sequence preceding Tyr132 (mouse numbering) we generated a *Lat*^NIL^ knock-in mouse strain in which the original sequence (coding for DADEDEDD) was replaced with an uncharged segment of equal length (GASGSNGN). Unexpectedly, homozygous *Lat*^NIL/NIL^ mice exhibited an almost complete block in thymic development at the DN3 stage and lacked mature CD4^+^ and CD8^+^ αβ T cells. RT-PCR analysis of thymocyte RNA revealed that the LAT mRNA was shorter than that expected for wild-type mice. Sequencing of LAT cDNA from *Lat*^NIL/NIL^ mice showed that exons 6 and 7 were deleted during RNA processing, resulting in a frameshift that introduced a premature STOP codon and produced a truncated LAT adaptor lacking the five distal tyrosine residues. Interestingly, *Lat*^NIL/NIL^ and LAT-KO mice exhibited a population of CD3^+^TCRγδ^+^ T cells in the spleen and lymph nodes, indicating that LAT-mediated signaling is not required for some forms of γδ T cell development. However, peripheral γδ T cells from *Lat*^NIL/NIL^ mice expressed higher levels of CD27 compared to those in wild-type and LAT-KO mice, suggesting that *Lat*^NIL/NIL^ mice may retain signaling capabilities that influence γδ T cell phenotype.

## 2. Results

### 2.1. Lat^NIL^ KI Mouse Generation

To investigate the in vivo role of the negatively charged amino acid segment of LAT between positions 124 and 131, which precedes the fifth tyrosine of LAT (position 132), we generated a knock-in (KI) mouse strain in which the aspartate and glutamate residues were replaced by glycine, serine or asparagine residues. These substitutions were designed based on previously published data regarding the substrate specificity of ZAP70, as this tyrosine kinase preferentially phosphorylates tyrosine residues preceded by glutamate or aspartate [20]. As shown in Supplementary Figure S1A, the stretch between residues 124 and 131 in mouse LAT contains seven negatively charged residues (aspartates or glutamates), which are evolutionarily conserved. The original sequence in mouse LAT is eight residues long with a net charge of −7 at pH 7, and we introduced a segment of eight amino acids with a neutral net charge (Supplementary Figure S1B).

In their work, Shah et al. analyzed the effect of exhaustive point-mutations of residues preceding tyrosine substrates of ZAP70, including Tyr127 (the human counterpart of mouse Tyr132). The substitutions introduced here confer flexibility to this fragment while having no effect on the phosphorylation of the adjacent tyrosine. The strategy used to engineer the present *Lat* mutant did not introduce any additional sequences and was limited to the intended mutations and a silent mutation introducing a new *Kpn*I site for detection of the recombinant allele (Supplementary Figure S1C). These minimal modifications were therefore expected to allow expression of the LAT^NIL^ mutant protein at levels comparable to those of wild-type LAT.

*Lat*^NIL^ KI mice were generated using a CRISPR/Cas9 nuclease-based approach on a C57BL/6 background (see Section 4), and mice homozygous for this mutation were born at expected Mendelian frequencies.

### 2.2. Thymic Development Arrest in Lat^NIL^ Mutant Mice

**Figure 1:**
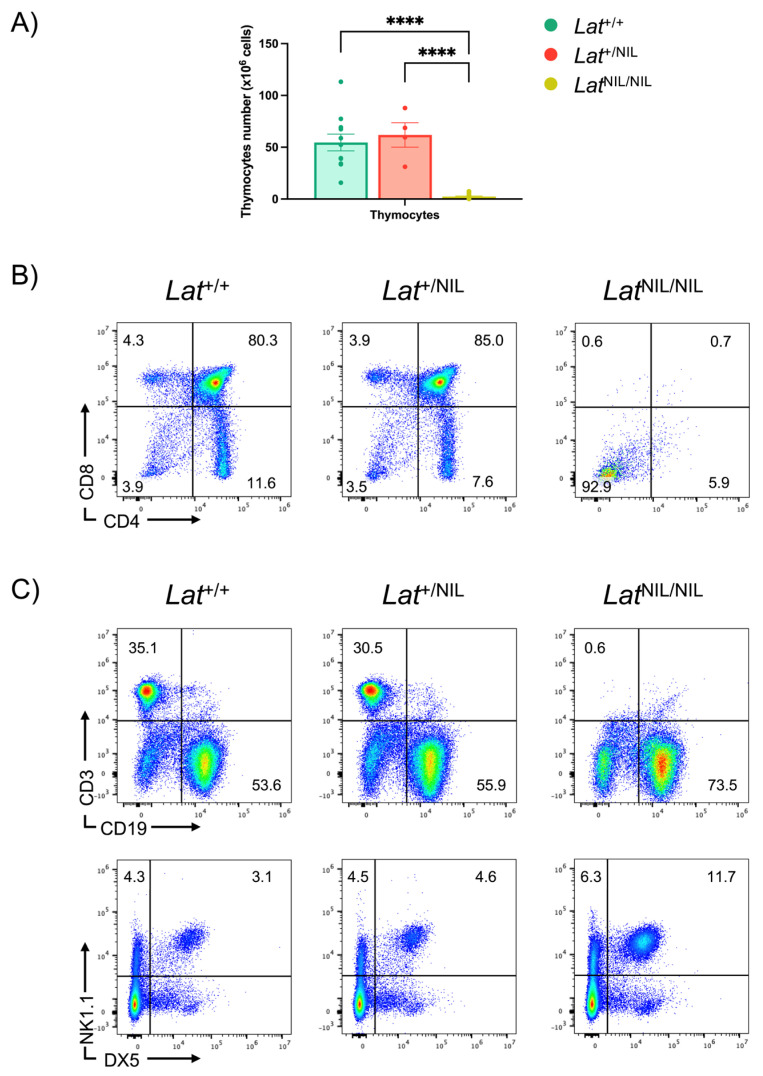
Flow cytometric analysis of thymic and splenic cells from *Lat*^+/+^, *Lat*^+/NIL^, and *Lat*^NIL/NIL^ mice. (**A**) Thymuses from 10- to 50-week-old adult *Lat*^+/+^ (n = 11), *Lat*^+/NIL^ (n = 4), and *Lat*^NIL/NIL^ (n = 19) mice were disaggregated and live thymocytes were counted. Bar graphs represent the mean values, and brackets on each bar represent the standard error mean. (**B**) Thymocytes from the three types of mice were surface stained and analyzed by standard flow cytometry. Two-color plots show staining of total thymocytes with antibodies against CD4 and CD8. Numbers in each quadrant represent the corresponding percentages. One representative experiment is shown. (**C**) Splenocytes from *Lat*^+/+^, *Lat*^+/NIL^, and *Lat*^NIL/NIL^ mice were surface stained with specific antibodies for CD3, CD19, NK1.1 and DX5. One representative experiment is shown. **** indicates *p* < 0.0001.

We examined the effects of the mutations introduced in *Lat*^NIL^ mice on thymic development. Heterozygous and homozygous *Lat*^NIL^ mice appeared healthy and fertile under specific pathogen-free conditions. Although the cellularity of the thymuses of heterozygous *Lat*^+/NIL^ mice showed no significant differences compared to those of wild-type thymuses, homozygous mutant mice exhibited a marked decrease in thymus size, with a mean number of 2.4 million cells, compared to 54.6 and 61.9 million thymocytes in *Lat*^+/+^ and *Lat*^+/NIL^ mice, respectively (Figure 1A). Flow cytometry analysis of thymocyte populations revealed that the homozygous *Lat*^NIL/NIL^ mutation completely blocked thymic development at the CD4^−^CD8^−^ double negative (DN) stage, resulting in the absence of CD4^+^CD8^+^ double-positive (DP), CD4^+^ single-positive (CD4^+^ SP), and CD8^+^ single-positive (CD8^+^ SP) cells in the thymus of *Lat*^NIL/NIL^ (Figure 1B). Consistent with the thymic phenotype, further analysis of the spleens of *Lat*^NIL/NIL^ mice showed a complete absence of T cells, whereas heterozygous mice exhibited normal T cell percentages (Figure 1C, upper panels). The percentage of natural killer cells was slightly increased in homozygous mutant mice compared to heterozygous and wild-type mice (Figure 1C, lower panels). Analysis of *Lat*^NIL/NIL^ lymph nodes yielded similar results, with a complete absence of T cells.

### 2.3. The Lat^NIL^ Mutation Generates an Alternative Splicing Event That Excludes Exons 6 and 7

**Figure 2:**
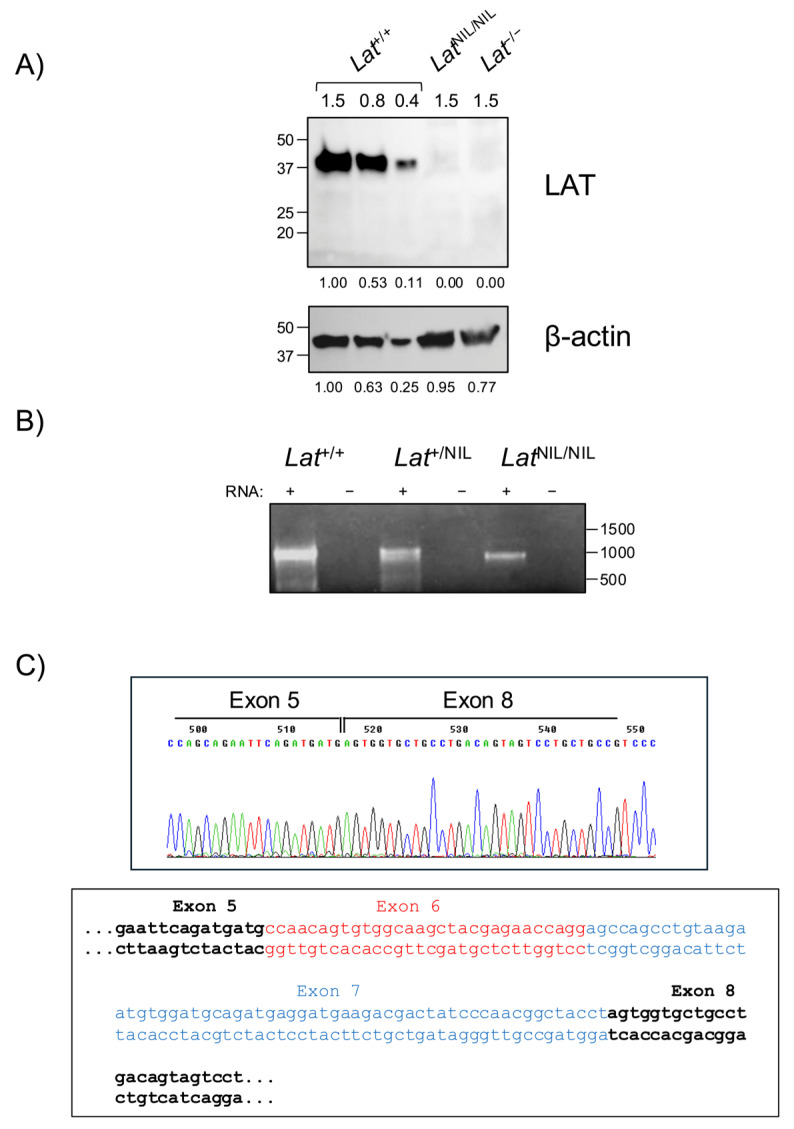
LAT expression in *Lat*^NIL/NIL^ mice. (**A**) Thymocytes from 18- to 26-week-old adult *Lat*^+/+^, *Lat*^NIL/NIL^, and *Lat*^−/−^ mice were lysed in Laemmli buffer, and samples corresponding to 1.5, 0.8 or 0.4 × 10^6^ cells were analyzed by Western blot for LAT expression (upper panel). The membrane was stripped and blotted with anti-β-actin antibody to show total protein load. A representative experiment out of three is shown. Numbers on the left indicate the approximate molecular weight (kDa). Numbers below each blot represent the densitometric quantification of individual bands. (**B**) Total RNA from 10-week-old adult *Lat*^+/+^, *Lat*^+/NIL^ and *Lat*^NIL/NIL^ was reverse transcribed, and the resulting cDNA was amplified with oligonucleotides spanning the entire LAT coding sequence. (**C**) Histogram showing the homozygous *Lat*^NIL/NIL^ cDNA sequence at the end of exon 5 (upper panel). The lower panel shows the wild-type LAT cDNA nucleotide sequence, including the coding regions for exons 6 (red) and 7 (blue), which are absent in *Lat ^NIL/NIL^* cDNA.

The total block we observed in homozygous *Lat*^NIL/NIL^ mice was unexpected, given the mild phenotype we had previously shown in J.CaM2 cells expressing a comparable LAT mutant [18], and was similar to the phenotype of LAT-KO and *Lat*^4YF^-knockin mice [9,10,11]. Therefore, we sought to verify LAT expression in homozygous *Lat*^NIL/NIL^ and wild-type mice. Thymocytes from wild-type and mutant mice were lysed and samples were analyzed by Western blot. Thymocytes from LAT-knockout (*Lat*^−/−^) mice were also included as a negative control. Lysates corresponding to 1.5, 0.8, and 0.4 × 10^6^ cells were analyzed with a rabbit monoclonal antibody specific for human and mouse LAT. This antibody was produced by immunizing animals with a synthetic peptide corresponding to residues around Leu88 of human LAT (Leu91 in mouse LAT). As shown in Figure 2A, LAT protein was not detected in the sample corresponding to homozygous *Lat*^NIL/NIL^ mice, as was the case in LAT-KO mice. This observation was quite surprising, given that the mutations introduced into LAT exon 7 were restricted to the coding region. To verify whether the defect in protein expression was due to a transcriptional defect, total RNA was extracted from thymocytes of wild-type, *Lat*^+/NIL^, and *Lat*^NIL/NIL^ mice, and PCR was performed on the cDNA obtained after the corresponding reverse transcription. Contrary to protein expression, thymocytes from homozygous *Lat*^NIL/NIL^ mice did express LAT mRNA, although the size appeared to be slightly shorter than that of WT mice (Figure 2B). Therefore, we purified PCR products from wild-type and *Lat*^NIL/NIL^ mice for sequence analysis. Supporting our previous observation, the mRNA from *Lat*^NIL/NIL^ mice was 91 bp shorter than that from wild-type mice, due to the removal of exons 6 and 7 during RNA splicing (Figure 2C).

This alternative splicing of mutant *Lat*^NIL^ RNA was an unexpected finding, since we did not change any nucleotide outside the coding region in exon 7. It has been previously reported that in humans a larger functional isoform of LAT can be expressed, which originates from an intron 6 retention event generating an in-frame splice variant of LAT mRNA denoted as LATi6 [21]. However, in the mouse *Lat* gene, retention of intron 6 is not possible because the size of that fragment is not a multiple of three (79 nucleotides), and its retention would introduce a premature stop codon close to the 5′ side of exon 7 (nucleotides 11 to 13). In *Lat*^NIL/NIL^ mice, the LAT protein that could be translated would consist of 145 amino acids. The elimination of 91 base pairs in exons 6 and 7 shifts the reading frame, introducing a premature stop codon, so that the N-terminal 106 amino acids remain identical to those of wild-type LAT (Supplementary Figure S2). This truncated form of LAT would lack the six C-terminal tyrosine residues, which accounts for the block in thymic development and the absence of peripheral T cells [9,11].

### 2.4. Marked Reduction in T Lymphocytes in Peripheral Lymphoid Organs of Homozygous Lat^NIL/NIL^ Mice

**Figure 3:**
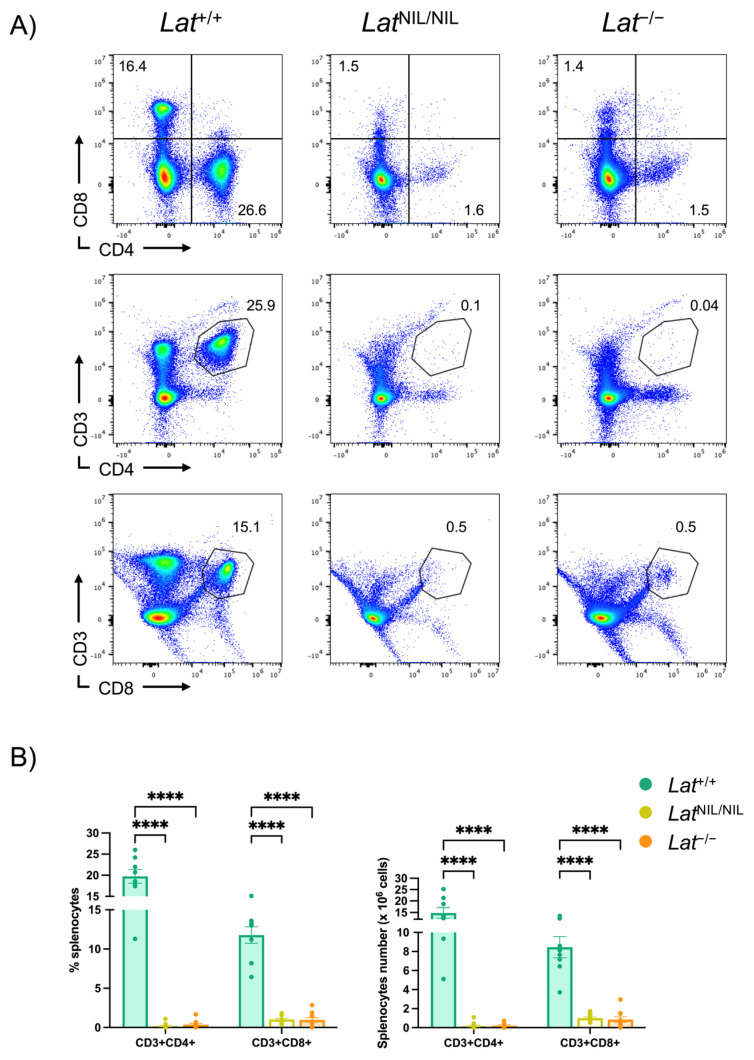
Effects of *Lat*^NIL^ mutation on peripheral lymphocyte populations. (**A**) Two-color plots show staining of total lymphocytes obtained from the spleens of the indicated strains of mice. The numbers included in each plot indicate the percentages of cells. One representative experiment is shown. (**B**) Bar graphs representing the mean values of CD3^+^CD4^+^ and CD3^+^CD8^+^ cell percentages (left panel) or total numbers (right panel) in the spleens of *Lat*^+/+^ (n = 11), *Lat*^+/NIL^ (n = 4), and *Lat*^NIL/NIL^ (n = 19) mice. **** indicates *p* < 0.0001.

The unforeseen alternative splicing observed in *Lat*^NIL/NIL^ mice prevented us from analyzing the in vivo role of the negatively charged residues in LAT preceding the fourth tyrosine in LAT. However, since no mouse model with a *Lat* gene coding for a truncated form of this adaptor had been previously generated, we decided to investigate the effects of this mutation on peripheral lymphoid populations. As expected, the *Lat*^NIL^ mutation, like LAT-KO, caused a marked decrease in CD4^+^ and CD8^+^ T cell populations in the spleen (Figure 3A, top panels) and lymph nodes (Supplementary Figure S3B). Interestingly, this absence was not total, with CD4^+^ T cell percentages of 1.0% and 1.3% for *Lat*^NIL/NIL^ and *Lat*^−/−^ mice, respectively, compared to 19.5% observed in wild-type mouse spleens (Supplementary Figure S3A). A similar finding was observed for CD8^+^ cells, with percentages of 1.9% and 2.0% for *Lat*^NIL/NIL^ and *Lat^−/−^*, respectively, compared to 13.5% observed in spleens from normal mice (Supplementary Figure S3A). Double CD3/CD4 and CD3/CD8 staining was performed to verify whether these populations corresponded to genuine T lymphocytes (Figure 3A, middle and bottom panels). In the case of CD4 T lymphocytes, the absence of CD3^+^CD4^+^ cells was nearly complete in *Lat*^NIL/NIL^ and *Lat*^−/−^ spleens (0.2% and 0.3%, respectively, Figure 3B). However, this was not the case for CD3^+^CD8^+^ cells, with mean percentages of 1.0% and 0.9% in spleens from *Lat*^NIL/NIL^ and *Lat*^−/−^ mice, corresponding to a mean number of 1.0 × 10^6^ and 0.9 × 10^6^ CD8 T cells per spleen of *Lat*^NIL/NIL^ and *Lat*^−/−^ mice, respectively (Figure 3B). Similar results were observed in peripheral lymph nodes (Supplementary Figure S3C,D). Therefore, these data led us to conclude that the *Lat*^NIL^ mutation causes an immunodeficiency in mice like that observed in LAT-KO mice, characterized by a near-total absence of CD4^+^ T cells, but not of CD8^+^ T cells. The latter population is present in both mouse strains, although in much lower numbers than in wild-type mice.

### 2.5. Effects of the Lat^NIL^ Mutation on γδ T Cells Development

**Figure 4:**
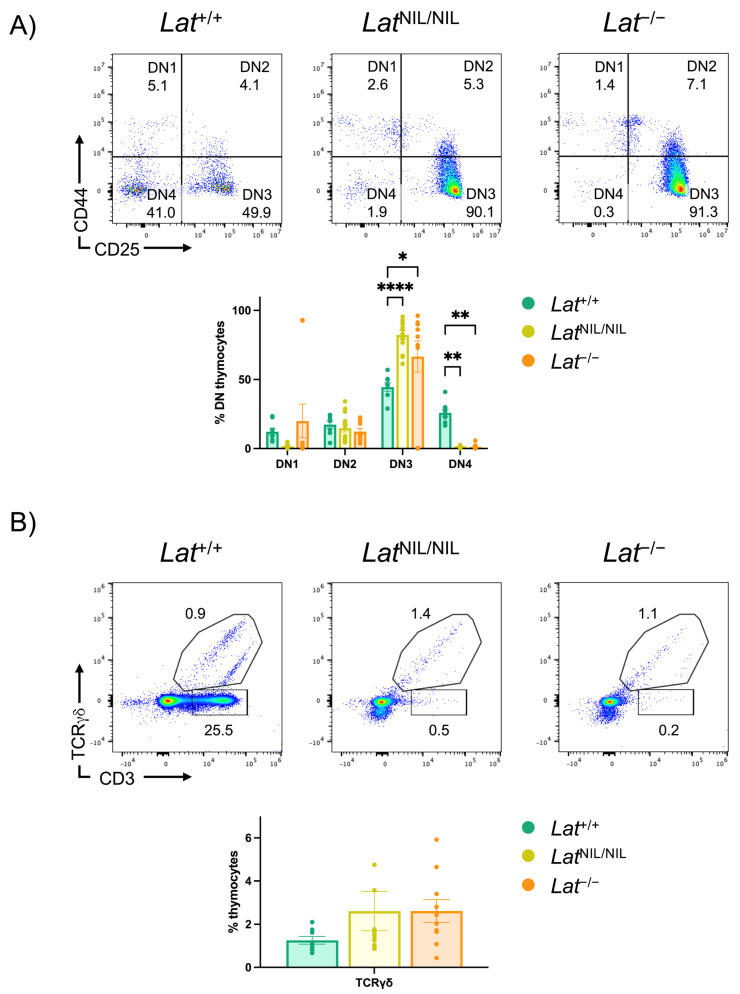
Impact of the *Lat*^NIL^ mutation on thymic development. (**A**) DN thymocytes (gated on CD3^−^ CD4^−^ CD8^−^ and B220^−^) were analyzed for the expression of CD44 and CD25 (top panels). Percentages of cells are shown in each quadrant. The lower bar graph represents the percentages of cells in each of the indicated compartments in wild-type (*Lat*^+/+^, n = 8), *Lat*^NIL/NIL^ (n = 13), and LAT-knockout (*Lat*^−/−^, n = 10) mutant mice. Brackets on each bar represent the standard error mean. (**B**) Analysis of CD3 and TCRγδ in total thymocytes. Percentages of cells are indicated next to each gate. The lower bar graph represents the percentages of cells in each of the indicated compartments. * indicates *p* < 0.05; ** indicates *p* < 0.01; **** indicates *p* < 0.0001.

**Figure 5:**
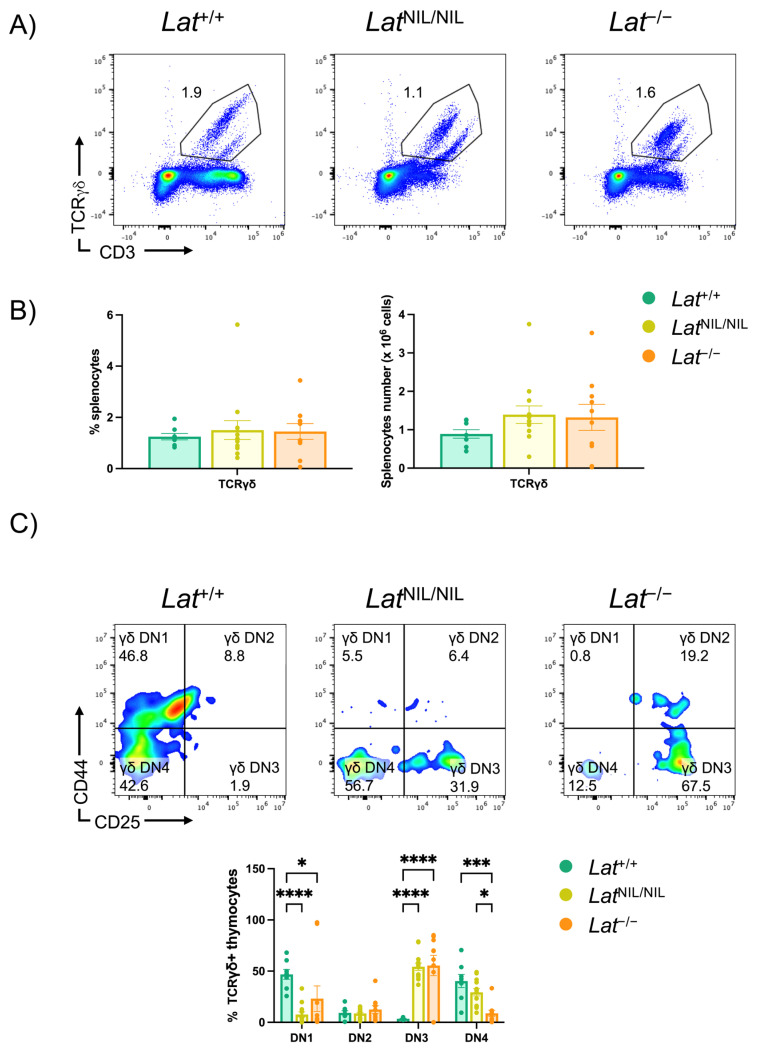
γδ T lymphocytes are found in the spleens of *Lat*^NIL/NIL^ and *Lat*^−/−^ mice. (**A**) Cytometric analysis of CD3 and TCR γδ expression in total splenocytes from 10 to 50 weeks old wild-type, *Lat*^NIL/NIL^, and *Lat*^−/−^ mutant mice. Percentages of cells are shown in each gate. (**B**) Bar graphs represent the percentages (left panel) and total number (right panel) of TCR γδ cells in the spleens from wild-type (*Lat*^+/+^, n = 8), *Lat*^NIL/NIL^ (n = 13), and LAT-knockout (*Lat*^−/−^, n = 10) mice. Brackets on each bar represent the standard error mean. (**C**) Analysis of the expression CD25 and CD44 in TCRγδ^+^ thymocytes from the indicated types of mice (upper panels). Percentages of cells are shown in each quadrant. The lower bar graph represents the percentages of cells in each of the indicated compartments. * indicates *p* < 0.05; *** indicates, *p* < 0.001; **** indicates *p* < 0.0001.

Initial reports suggested that the absence of LAT completely blocks thymic development at the DN stage, thereby preventing γδ T cell development [9,10,11]. However, a recent study showed that about 25% of LAT-knockout DN3 γδ thymocytes were in the S or G2/M phases of the cell cycle, and that 30% of LAT-deficient DN3 γδ cells expressed CD71, a hallmark of pre-TCR and γδ-TCR signaling [22,23]. Based on these observations, we examined early thymic development in *Lat*^NIL/NIL^ and *Lat*^−/−^ mice and confirmed that thymocyte development was arrested at the DN3 stage in LAT-KO mice, consistent with the phenotype observed in *Lat*^NIL/NIL^ mice (Figure 4A). Analysis of γδ TCR and CD3 expression in total thymocytes showed similar percentages of γδ T cell progenitors in both *Lat*^NIL/NIL^ and *Lat*^−/−^ mice (Figure 4B), and we did not observe any statistically significant differences in the percentage of γδ T cells between any of the three types of mice analyzed. As expected, the population of CD3^+^ γδ TCR^−^ cells (potentially the precursors of conventional αβ T cells) was virtually absent in *Lat*^NIL/NIL^ and *Lat*^−/−^ mice, which was consistent with previous data and with the blockade at the DN3 stage. However, contrary to what had been previously described in LAT-KO and *Lat*^4YF^ mice [9,10,11], our analyses of *Lat*^NIL/NIL^ and *Lat*^−/−^ mice revealed the presence of γδ T cells in peripheral lymphoid organs (Figure 5A,B). These γδ T cell populations observed in *Lat*^NIL/NIL^ and *Lat*^−/−^ mice were different from the TH2-type γδ T cells that accumulated in large numbers in peripheral lymphoid organs of *Lat*^3YF^ mice [10]. *Lat*^3YF^ mice, carrying Tyr-to-Phe substitutions at the three C-terminal tyrosine residues of LAT, exhibited a marked accumulation of γδ T cells from 20 weeks of age onward. These cells proliferated continuously in a spontaneous manner and displayed an activated phenotype. We analyzed mice up to 50 weeks of age, and in no case did the number of γδ T lymphocytes exceed 4 million cells in the spleens of *Lat*^NIL/NIL^ or *Lat*^−/−^ mice (Figure 5B). γδ T cells were also present in the lymph nodes of both types of mutant mice, with percentages that were in no case significantly higher than those of wild-type mice (Supplementary Figure S4A,B).

Given the unexpected presence of γδ T lymphocytes in the peripheral lymphoid organs of *Lat*^NIL/NIL^ and *Lat*^−/−^ mice, we investigated whether their thymic maturation was completely blocked. Most γδ TCR-expressing thymocytes lacked expression of both CD4 and CD8 (γδ DN), and the proportion of this population was significantly higher in *Lat*^NIL/NIL^ and *Lat*^−/−^ mice compared to wild-type control mice (Supplementary Figure S4C,D). Consistently, the frequency of γδ CD4^+^CD8^+^ thymocytes (γδ DP) was reduced in *Lat*^NIL/NIL^ and *Lat*^−/−^ mice relative to wild-type mice, suggesting a developmental block. Interestingly, recent single-cell RNA sequencing (scRNA-seq) analyses have identified, within the DN4 thymocyte compartment of *Lat*^−/−^ mice, a minor population of γδ TCR-expressing cells [23], suggesting that the developmental block affecting this cell subset in the absence of LAT is not as strict as previously thought. We therefore examined CD44 and CD25 expression in γδ TCR^+^ thymocytes, as the expression of this receptor has previously been demonstrated in stages as early as DN1 [24]. Although stages DN1 and DN2 are common to both αβ and γδ T cell development, we sought to determine whether γδ T cell development is blocked in *Lat*^NIL/NIL^ or *Lat*^−/−^ mice. In wild-type mice, the majority of γδ TCR^+^ thymocytes (46.9%) were γδ DN4 cells, suggesting that they had already been selected to become γδ T lymphocytes (Figure 5C). However, the CD44/CD25 expression pattern differed in *Lat*^NIL/NIL^ and *Lat*^−/−^ mice, with a clear accumulation of γδ DN3 cells and a large decrease in γδ DN4 cells, which closely resembles what occurs in total DN thymocytes in *Lat*^−/−^ mice (Figure 4A). Interestingly, the block in γδ T cell development appeared less stringent in *Lat*^NIL/NIL^, as the percentage of γδ DN4 cells was significantly higher than in *Lat*^−/−^ mice (Figure 5C, lower panel). Thus, the *Lat*^NIL^ mutation, which potentially generates a truncated form of LAT, may have different effects than LAT knockout on γδ T cell development.

### 2.6. Phenotype of γδ T Cells Found in Lat^NIL/NIL^ and Lat^−/−^ Mice

**Figure 6:**
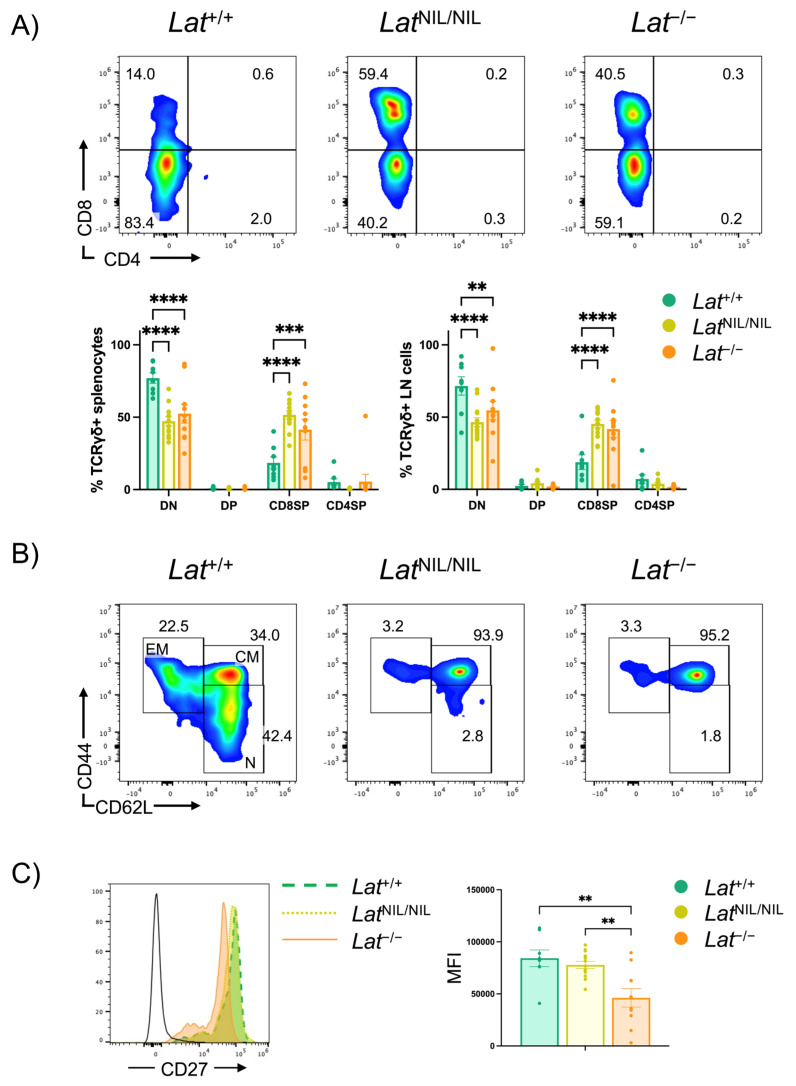
Phenotype of γδ T lymphocytes from *Lat*^NIL/NIL^ and *Lat*^−/−^ mice. (**A**) CD4 and CD8 expression analysis in CD3^+^TCRγδ^+^ splenocytes from wild-type, *Lat*^NIL/NIL^, and *Lat*^−/−^ mutant mice. Percentages of cells are shown in each quadrant (upper panels). Lower bar graphs represent the percentages of TCR γδ cells in the spleens (left panel) and lymph nodes (right panel) from wild-type (*Lat*^+/+^, n = 8), *Lat*^NIL/NIL^ (n = 13), and LAT-knockout (*Lat*^−/−^, n = 10) mice. Brackets on each bar represent the standard error mean. (**B**) Analysis of memory and naive γδ T cells in spleen from wild-type, *Lat*^NIL/NIL^, and LAT-knockout mice. The numbers in the depicted regions represent the percentage of cells. EM stands for Effector Memory; CM stands for Central Memory; and N stands for Naive. A representative experiment is shown. (**C**) Analysis of CD27 expression in CD3^+^TCRγδ^+^ splenocytes from wild-type, *Lat*^NIL/NIL^, and *Lat*^−/−^ mutant mice (left panel). The bar graph (right panel) represents the mean value of the Mean Fluorescence Intensity of CD27 in γδ T cells from the spleens of wild-type, *Lat*^NIL/NIL^, and *Lat*^−/−^ mutant mice. Black line histogram indicate staining with an isotype-matched negative control antibody. ** indicates *p* < 0.01; *** indicates, *p* < 0.001; **** indicates *p* < 0.0001.

Next, we sought to analyze the phenotype of γδ T cells found in the spleens of *Lat*^NIL/NIL^ and *Lat*^−/−^ adult mice. CD4/CD8 staining revealed that the majority of splenic γδ T cells (77.1% ± 3.6) from wild-type mice did not express either CD4 or CD8 (Figure 6A). In contrast, this double-negative population was markedly reduced in *Lat*^NIL/NIL^ and *Lat*^−/−^ mice (47.3% ± 2.9 and 52.4% ± 6.4, respectively), accompanied by a significant increase in the percentage of γδ T cells expressing CD8 (Figure 6A). The frequency of CD8^+^ γδ T cells did not differ significantly between *Lat*^NIL/NIL^ and *Lat*^−/−^ mice in the spleen or lymph nodes (Figure 6A and Supplementary Figure S5A).

We also examined the activation status of γδ T cells in LAT mutant mice, as the γδ T cell population that expands in the spleens and lymph nodes of *Lat*^3YF^ mice exhibits a CD44^hi^CD62L^low/neg^ phenotype [10]. Surprisingly, most γδ T cells from the spleens and lymph nodes of *Lat*^NIL/NIL^ and *Lat*^−/−^ mice displayed a CD44^hi^CD62L^hi^ phenotype, characteristic of central memory T cells (Figure 6B and Supplementary Figure S5B). Consequently, the proportions of naive (CD44^low/neg^CD62L^hi^) and effector memory (CD44^hi^CD62L^low/neg^) cells were reduced in both *Lat*^NIL/NIL^ and *Lat*^−/−^ mutant mice, although these differences did not reach statistical significance. As shown in Supplementary Figure S5B, no differences were observed between *Lat*^NIL/NIL^ and *Lat*^−/−^ mice in any of these populations.

Finally, we analyzed the CD27 expression in γδ T cells from the spleens and lymph nodes of *Lat*^NIL/NIL^ and *Lat*^−/−^ mutant mice. In wild-type mice, most γδ T cells acquire a functional polarization, with CD27 serving as a marker of IFN-γ-producing (CD27^+^) γδ T cells, whereas CD27^−^ γδ T cells primarily secrete IL-17 [25]. As shown in Figure 6C, the majority of splenic γδ T cells expressed CD27, and no statistically significant differences were observed in the proportion of CD27^+^ γδ T cells among the three genotypes analyzed. A similar pattern was observed in lymph node γδ T cells (Supplementary Figure S5C, upper panel). However, analysis of CD27 expression intensity revealed that γδ T cells from wild-type and *Lat*^NIL/NIL^ mutant mice exhibited significantly higher levels than those from LAT-knockout mice (Figure 6C, right panel). The same trend was observed in the analysis of lymph node cells (Supplementary Figure S5C, upper panel), although statistical significance was not reached in this case (*p* = 0.165). Together, these results indicate that LAT is not essential for γδ T cell development and suggest that the truncated form of LAT generated by the *Lat*^NIL^ mutation may retain residual signaling capabilities.

## 3. Discussion

The LAT adaptor protein plays an essential role in transducing signals from the TCR, which are crucial for the development, activation, and differentiation of T cells [26,27]. To carry out its functions, LAT depends on a series of evolutionarily conserved tyrosine residues, which, after phosphorylation, become binding sites for other cytosolic proteins [28]. This leads to the assembly of the LAT signalosome, for which the proper phosphorylation kinetics of the different tyrosine residues is essential. In this context, it has been demonstrated that phosphorylation of the sixth tyrosine of LAT (residue 136 in mouse) is of critical relevance for T cell ligand discrimination after TCR-pMHC interaction [29,30,31]. Interestingly, the sixth tyrosine of LAT plays a dual role in the TCR signaling cascade, since after its phosphorylation it binds to PLC-γ1, allowing its activation and the subsequent increase in cytosolic calcium concentration [7,8,13]. However, its mutation to phenylalanine in mice generates a lymphoproliferative disorder in homozygous mutant mice, revealing a crucial role for this residue in regulating T cell homeostasis [12]. This tyrosine has the special feature of being preceded by a non-charged glycine residue, unlike other functionally relevant tyrosines of LAT (located in positions 175, 195, and 235, mouse numbering), which are preceded by negatively charged residues (Glu or Asp). As shown by Kuriyan and co-workers, ZAP70 selects its substrates by utilizing an electrostatic mechanism that favors LAT and SLP-76 tyrosine residues preceded by negatively charged amino acids [20,32]. Consequently, the glycine residue preceding the sixth tyrosine in LAT appears to represent a critical bottleneck in TCR signaling, constraining the TCR-induced activation of PLCγ1 and the downstream calcium signaling pathways [27]. Mutation of this glycine residue to aspartate accelerates Tyr136 phosphorylation, altering thymic development, increasing the fraction of memory T cells, and disrupting ligand discrimination [29,30,31]. Therefore, negatively charged amino acids before LAT tyrosine residues seem to be important for rapid phosphorylation and efficient assembly of the LAT signalosome.

In this context, we had previously shown that substitution of an evolutionarily conserved segment of negatively charged residues preceding Tyr127 in human LAT (Tyr132 in mouse numbering) induced an increase in proximal intracellular signals but also reduced downstream signals such as Ca^2+^ influx or Erk activation [18]. Also of interest, aspartates can be targets for caspase proteases, and we had previously shown that LAT is proteolytically cleaved at three aspartate residues, one of them the one preceding Tyr132 of mouse LAT [33]. Moreover, induction of LAT tyrosine phosphorylation inhibited its proteolytic cleavage. Therefore, to investigate the physiological role of this stretch of negatively charged amino acids, we generated a knock-in strain (*Lat*^NIL^ KI) with a segment of the same length, but without charge (GASGSNGN). Unexpectedly, homozygous *Lat*^NIL/NIL^ mice showed a severe block in thymic development, with a large reduction in thymic cellularity, the absence of DP and SP thymocytes, and peripheral lymphoid organs devoid of conventional CD4 and CD8 T lymphocytes. This phenotype was surprising given that previous data obtained in J.CaM2 cells expressing a similar LAT mutant did not block TCR signals. Indeed, Western blot analysis of thymocytes from homozygous *Lat*^NIL/NIL^ mice demonstrated a complete loss of LAT protein expression. This was the result of an unexpected alternative splicing event removing exons 6 and 7 from the mRNA, generating a frameshift and, consequently, a premature stop codon. The predicted protein in T cells from homozygous *Lat*^NIL/NIL^ mice would contain 145 amino acids, with the N-terminal 106 residues matching those of the wild-type protein.

Model exon studies have demonstrated that exon skipping can result from mutations distributed throughout the exon in some cases, whereas in others, only mutations near the splice sites exert an effect [34]. It has been shown that the first and last three exonic positions are integral components of the 3′ and 5′splice site (3′ss and 5′ss) consensus sequences [35]; however, none of these nucleotides were modified in exon 7 of the *Lat* gene (Supplementary Figure S1). Exons also contain splicing regulatory elements, such as exonic splicing enhancers (ESEs) and exonic splicing silencers (ESSs) [36,37]. We performed a comparative analysis of exon 7 sequences in wild-type and *Lat*^NIL^ using the ESEfinder tool (https://esefinder.ahc.umn.edu/) [38], which revealed that two putative binding sites for the serine/arginine-rich splicing factors SRSF1 and SRSF2 were lost due to the mutations introduced in *Lat*^NIL^ (Supplementary Figure S6). This may explain the aberrant splice-site recognition. Moreover, the substitutions introduced in *Lat*^NIL^ generated a new potential binding site for SRSF5, which has been reported to promote both exon inclusion [39,40] and exon skipping [41]. Regardless of the precise cause of this alternative splicing event, it prevented us from examining the effect of the intended mutations in the negatively charged segment preceding Tyr132 in mouse LAT. LAT plays an important role in balancing intracellular T cell activation signals. Our objective was to analyze the physiological functions of the negatively charged residues upstream of Tyr132 in mouse LAT during TCR activation and in the interaction of LAT with other signaling molecules. Although CRISPR/Cas9 technology enables the rapid and accessible generation of knock-in mice compared with conventional ES cell technology, a classic strategy involving the introduction of a mutated LAT minigene (to bypass splicing) would have avoided this problem.

Our work further demonstrates that the absence of LAT does not completely prevent the development of γδ T cells. Early studies using LAT-knockout mice reported the presence of γδ T cells in the thymus; however, these cells were absent in peripheral lymphoid organs [9,10]. Our initial analyses comparing *Lat*^NIL/NIL^ mice with wild-type controls revealed the presence of γδ T cells in the spleen and lymph nodes, suggesting that *Lat*^NIL/NIL^ mice exhibit immune features similar to those observed in *Lat*^3YF^ mice [10]. Therefore, the absence of LAT expression, or expression of a truncated form lacking critical tyrosine residues, could promote the proliferation of γδ T cells that have bypassed thymic development. Notably, spleens from homozygous *Lat*^NIL/NIL^ or LAT-knockout (*Lat*^−^/^−^) mice contained no more than 4 million cells, far fewer than the ~100 million γδ T cells observed in *Lat*^3YF^ mice [10]. Comparative analysis of *Lat*^NIL/NIL^ and *Lat*^−/−^ mice confirmed that γδ T cell development is LAT-independent, as both strains exhibited similar cell numbers in the spleen and lymph nodes. This finding aligns with a previous work on γδ T cell commitment during thymic development [23]. Approximately 25% of DN3 γδ thymocytes from *Lat*^−/−^ mice were in S and G2/M phases, compared to 79% in wild-type DN3 γδ thymocytes, indicating that in the absence of LAT, the γδ TCR can still deliver LAT-independent signals sufficient to drive a subset of cells into the cell cycle. Additionally, a similar proportion of DN3 γδ thymocytes from *Lat*^−/−^ mice expressed CD71, a marker of TCR signaling [22], and small numbers of DN4 γδ cells were observed. Interestingly, it was previously shown that in a LAT-deficient mouse strain (*Lat*^Inv/Inv^) there was an accumulation of Thy1^+^ T cells, and some of them exhibited non-productive TCR-γ gene rearrangements [42]. In the present study, although *Lat*^NIL/NIL^ and *Lat*^−/−^ mice exhibited accumulation of γδ DN3 (CD44^−^CD25^+^) cells, a population of γδ DN4 (CD44^−^CD25^−^) cells was present in both strains, indicating that γδ T cell development is not completely arrested in the absence of LAT. Interestingly, the proportion of γδ DN4 cells was significantly higher in *Lat*^NIL/NIL^ mice than in *Lat*^−/−^ mice, potentially reflecting residual signaling capacity of a truncated LAT form. Western blot analysis using a rabbit monoclonal antibody against residues surrounding Leu88 of human LAT (a region conserved in mouse LAT) did not detect a truncated LAT protein in *Lat*^NIL/NIL^ thymocytes. Proteomic analysis identified several LAT peptides in wild-type samples, but none were detected in *Lat*^NIL/NIL^ lysates. Further studies are needed to determine whether a truncated LAT form is transiently expressed in *Lat*^NIL/NIL^ thymocytes, which may explain the differences observed relative to *Lat*^−/−^ mice. It is possible that the truncated form of LAT that would be generated in these mice is inherently unstable. Supporting this, expression analyses of LAT truncates in Jurkat cells revealed stable expression of only the 186-residue form, while the 126- and 138-residue truncates were undetectable or expressed at very low levels. In this context, it has been shown that LAT is ubiquitinated upon TCR activation, triggering its degradation and limiting the duration of TCR signaling [43]. One possible explanation for the failure to detect the truncated LAT variant is that the conserved lysine 52 site, combined with the absence of key protein interactions, may render it particularly unstable, resulting in rapid degradation.

It is also noteworthy that γδ T cells present in the spleens and lymph nodes of *Lat*^−^/^−^ and *Lat*^NIL/NIL^ mice displayed a phenotype markedly distinct from that observed in γδ cells from wild-type mice. Consistent with previous reports [44], our data indicate that the majority of γδ cells from wild-type mice did not express either CD4 or CD8 (77.1% ± 3.6), while a minor CD8^+^ population was detected (18.5% ± 4.1). In contrast, *Lat*^NIL/NIL^ and *Lat*^−/−^ mice exhibited a significant increase in the proportion of γδ T cells expressing CD8 (51.6% ± 2.8 and 41.4% ± 7.1, respectively), accompanied by a concomitant reduction in the CD4^−^CD8^−^ double-negative subset. Notably, previous studies have shown that the CD8^+^ subset of mouse γδ T cells proliferates rapidly under lymphopenic conditions [45], which could explain the prevalence of this subset in the lymphopenic environment of *Lat*^NIL/NIL^ and *Lat*^−/−^ mice. The proportion of CD8^+^ cells was somewhat higher in *Lat*^NIL/NIL^ compared to LAT-knockout mice; however, this difference did not reach statistical significance.

Of interest, most peripheral γδ T cells from *Lat*^NIL/NIL^ and *Lat*^−/−^ mice displayed a central memory phenotype (CD44^hi^CD62L^hi^), with proportions significantly higher than those observed in wild-type mice. Finally, similar to wild-type mice, most γδ T cells from *Lat*^NIL/NIL^ and LAT-knockout mice expressed CD27, a marker of functional polarization to a γδT1 (IFN-γ-producing) phenotype [25,46]. Although it has been proposed that stronger TCR signals promote γδT1 cell differentiation [47], our data do not support this model, as *Lat*^NIL/NIL^ and *Lat*^−/−^ mice (with severely compromised TCR signaling) showed no evident differences in the proportions of CD27^+^ γδ T cells compared to wild-type mice. However, the intensity of CD27 expression was significantly higher in γδ T cells from the spleens of wild-type and *Lat*^NIL/NIL^ mutant mice compared to those from LAT-knockout mice, and a similar trend was observed in γδ lymph node cells. We cannot exclude that residual signaling in γδ T cells from *Lat*^NIL/NIL^ mice accounts for these subtle differences between *Lat*^NIL/NIL^ and *Lat*^−/−^ mice. Further work is needed to clarify these questions, but our findings suggest that LAT-dependent signaling partially modulates γδ T cell subset distribution and functional maturation. Nonetheless, certain aspects of γδ T cell differentiation, including CD27 expression, appear to occur independently of robust TCR signaling.

## 4. Materials and Methods

### 4.1. Antibodies and Reagents

Anti-CD3, -CD4, -CD8, -CD25, -CD27, -CD44, -CD49b (DX5), -CD62L, -CD69, -CD73, NK1.1, -TCRβ and -TCRγδ, and their corresponding isotype controls were obtained from BioLegend (San Diego, CA, USA). Antibodies specific for LAT (ref. 45533) and β-Actin were purchased from Cell Signaling Technologies (Danvers, MA, USA). Prior to antibody staining, a live/dead viability kit (Molecular Probes, Cat. L34967, Thermo Fisher Scientific, Rochester, NY, USA) was used to assess cell viability, and the Fc receptors were blocked with Trustain FCX from BioLegend. Cells were then stained for 30 min on ice, washed twice in phosphate-buffered saline containing 1% fetal bovine serum, and analyzed on a Cytek^®^ Aurora 5L 16UV-16V-14B-10YG-8R spectral cytometer (Cytek Biosciences, Fremont, CA, USA).

### 4.2. Animals

Mice were maintained and used at the local Animal Supply Services (SEPA, University of Cadiz). Animal care and handling followed the guidelines of the European Union Council (2010/63/EU, 86/609/UE) on the use of laboratory animals. Experimental procedures were approved by the local Animal Care and Ethics Committee (University of Cadiz, Cadiz, Spain) and the Ministry of Agriculture, Fisheries and Rural Development (Junta de Andalucía, Spain).

LAT-knockout (*Lat*^−/−^) mice were generated as previously described by means of a classical approach involving Embryonic Stem (ES) cells [10]. This strategy removed a 5353 bp fragment comprising the entire LAT coding region. *Lat*^−/−^ mice were backcrossed onto C57BL/6J mice for more than 10 generations.

The number and age of the animals used for each experiment are provided in the corresponding figure captions throughout the manuscript. The weight of the animals depended on their sex and age and was always between 16 and 34 g. No significant differences were found in the weight of animals of different genotypes.

### 4.3. Generation of Knockin Mouse Expressing a LatNIL Allele

A CRISPR/Cas9 nuclease approach was adopted for direct mutagenesis of *Lat* exon 7 sequence using an ssODN template harboring the desired mutations. To generate the knock-in allele, we employed a CRISPR/Cas9 ribonucleoprotein (RNP)-based genome-editing strategy combined with zygote electroporation, as detailed below. A chimeric single guide RNA (sgRNA) targeting the sequence 5′-GATGAAGACGACTATCCCAA***CGG***-3′ (the protospacer adjacent motif (PAM) is shown in italics and bold) found in exon 7 of the *Lat* gene was synthesized. A single-stranded deoxynucleotide (ssODN) was designed which incorporates the desired knock-in mutations, together with a silent mutation to generate a unique *Kpn*I restriction site, allowing for easy detection of the recombinant allele.

The 139 nucleotides long Lat-NIL template (5’-TCCAGGTCGTGTTAACTCTCCTTTCTCACAGAGCCAGCCTGTAAGAATGTGG**G**TGCA**TC**TG**G**G**TC**T**A**A**T**G**G**C**A**ACTATCCCAACGG**G**TACCTGTGAGTGGGTAGAGGGGAGGTGACCGTGGAAGTTGTGTGCCCTTTAT-3′; mutated nucleotides are shown underlined and bold) was synthesized as a ssDNA and purified using polyacrylamide gel electrophoresis. Three-week-old C57BL/6J female mice were superovulated and mated with C57BL/6J male mice, and embryos were flushed from the uteri of successfully mated mice on day 0.5 of pregnancy and cultivated for 2–3 h until most obtained embryos had two clear pronuclei visible. For genome editing, an electroporation mix containing sgRNA (136 ng/µL), Cas9 nuclease (650 ng/µL; IDT), and 10 µM ssODN in OptiMEM was prepared to generate the Cas9–sgRNA RNP complex together with the donor template. Zygotes were then incubated in this media and electroporated on a 1mm electroporation slide (2 square-wave pulses of 30 V, 3 ms duration and 100 ms interval) following by overnight incubation. The resulting two-cell embryos were then reimplanted into the oviduct of pseudopregnant CD1 foster mothers at 0.5 days post-coitum.

Genomic DNA was isolated from the tails of the resulting F0 mice, and a 510-bp region encompassing exon 7 was amplified using the following PCR primers: 5′-GGGCTTTGGGGAGGATGTAC-3′ (Fwd) and 5′-GAAATGGAAGGGCGGCAATC-3′ (Rev). The PCR product was digested with *Kpn*I to assess whether the restriction site had been introduced into the genome. The wild-type *Lat* amplicon remains undigested (501 bp), whereas homologous recombination with the ssODN donor generates a *Kpn*I restriction site, producing two fragments of 230 bp and 271 bp. Indel mutations generated by non-homologous end-joining were detected based on shifts in amplicon size on agarose gels.

### 4.4. Preparation of Cell Lysates and Western Blotting

Thymocytes from wild-type or mutant mice were lysed at 1.0 × 10^8^ cells/mL in 2× Laemmli buffer, followed by incubation at 95 °C for 5 min and sonication. For Western blotting, whole-cell lysates were separated by SDS-PAGE and transferred to PVDF membranes, which were incubated with the listed primary antibodies, followed by the appropriate secondary antibody conjugated to IRDye 800CW (Li-Cor, Lincoln, NE, USA) or horseradish peroxidase (HRP). Reactive proteins were visualized using the Odyssey CLx Infrared Imaging System (Li-Cor) or by enhanced chemiluminescence (ECL) acquired in a ChemiDoc Touch Imaging System (Bio-Rad Laboratories, Hercules, CA, USA). For reprobing, PVDF membranes were incubated for 10 min at room temperature with WB Stripping Solution (Nacalai Tesque, Kyoto, Japan), followed by a TTBS wash. Western blots were densitometrically quantified, and statistics were performed with GraphPad Prism, version 10.5.0.

### 4.5. RNA Isolation, cDNA Synthesis and PCR

Cytoplasmic RNA was purified from thymocytes using TRIzol™ LS Reagent (Invitrogen, Thermo Fisher Scientific, Waltham, MA, USA) according to the manufacturer’s protocol. RNA was reverse transcribed using qScript™ cDNA Synthesis kit (QuantaBio, Beverly, MA, USA) and used for conventional PCRs with DreamTaq DNA Polymerase (Fermentas, Thermo Fisher Scientific). Primer sequences for mouse LAT PCR were: 5′-CGAAAGGATACGGCTGCCTACT-3′ and 5′-CCCAGCAAGTCCAGTTTCAGGA-3′.

### 4.6. Statistical Analysis

Statistics were performed with GraphPad Prism using one-way or two-way ANOVA tests, using an alpha level of 0.05 and 95% confidence intervals. Tukey’s HSD post hoc test was applied for multiple comparisons following significant ANOVA results. Levels of significance *p* < 0.05 are presented as *, *p* < 0.01 as **, *p* < 0.001 as *** and *p* < 0.0001 as ****.

## 5. Conclusions

Our study reveals that attempts to replace the negatively charged segment preceding Tyr132 of LAT in vivo unexpectedly triggered an alternative splicing event that abolished expression of the full-length adaptor protein, preventing assessment of the intended physiological function of this region. The resulting *Lat*^NIL/NIL^ mice exhibited a profound developmental block in the αβ T cell lineage, consistent with the essential role of LAT in thymocyte maturation and TCR signal transduction. Despite the absence of detectable LAT protein, γδ T cell development was not completely arrested, demonstrating that key aspects of γδ T cell differentiation can occur independently of LAT-dependent signaling. Moreover, *Lat*^NIL/NIL^ and Lat^−/−^ mice shared similar γδ T cell numbers, subset distributions, and memory phenotypes, although subtle differences may reflect residual or transient expression of a truncated LAT variant. Together, these findings underscore the strict requirement for LAT in αβ T cell development, highlight LAT-independent pathways that contribute to γδ T cell maturation, and emphasize the importance of considering unintended splicing outcomes when applying CRISPR/Cas9-mediated genome editing to genes with complex regulatory architecture.

## Data Availability

The original contributions presented in this study are included in the article and Supplementary Materials. Further inquiries can be directed to the corresponding author.

## Supplementary Materials

The following supporting information can be downloaded at: https://www.mdpi.com/article/10.3390/ijms262412186/s1.

## Abbreviations

The following abbreviations are used in this manuscript:

Erk	Extracellular Signal-Regulated Kinase
Gads	Growth factor receptor-bound 2-related adaptor downstream of Shc
Grb2	Growth factor receptor-bound 2
LAT	Linker for the Activation of T cells
PLC-γ1	Phospholipase C-γ1
TCR	T Cell Receptor
