# Supplementary material for: Low Levels of Mouse γδ T Cell Development Persist in the Presence of Null Mutants of the LAT Adaptor
Source: Int J Mol Sci. 2025 Dec 18;26(24):12186. doi: 10.3390/ijms262412186 (PMC12734389; doi:10.3390/ijms262412186)
Supplement: Supplementary file 1 [file ijms-26-12186-s001.zip › ijms-4015053-supplementary.pdf]

Supplemental Figures

Low Levels of Mouse γδ T Cell Development Persist in the Presence of Null Mutants of the LAT Adaptor

Mikel M. Arbulo-Echevarria [1,2,†], Luis M. Fernandez-Aguilar [1,2], Elke Kurz [3], Inmaculada Vico-Barranco [1,2], Raquel Muñoz-Fernández [1,2], Isaac Narbona-Sánchez [1,2], Manuel Carrasco [1,2], Bernard Malissen [4,5,6], Michael L. Dustin [3,7] and Enrique Aguado [1,2,*]

1. Department of Biomedicine, Biotechnology and Public Health, Universidad de Cádiz, 11002 Cádiz, Spain.

2. Biomedical Research and Innovation Institute of Cádiz (INiBICA), 11009 Cádiz, Spain.

3. Kennedy Institute of Rheumatology, Nuffield Department of Orthopaedics, Rheumatology and Musculoskeletal Sciences, University of Oxford, Oxford OX1 7FY, UK.

4. Centre d'Immunologie de Marseille-Luminy (CIML), Aix Marseille Université, Institut National de la Santé et de la Recherche Médicale (INSERM), Centre National de la Recherche Scientifique (CNRS), 13009 Marseille, France.

5. School of Medical Technology, Henan Medical University, Xinxiang 453003, China.

6. Centre d'Immunophénomique (CIPHE), Aix Marseille Université, Institut National de la Santé et de la Recherche Médicale (INSERM), Centre National de la Recherche Scientifique (CNRS), 13009 Marseille, France.

7. Chinese Academy of Medical Science Oxford Institute, Nuffield Department of Medicine, University of Oxford, Oxford OX3 7BN, UK.

* Correspondence: enrique.aguado@uca.es

† Current address: Molecular Oncology Group, Biogipuzkoa Health Research Institute, 20014 San Sebastián, Spain

A)

Mus musculus	NQEPACKNVDADEDEDDYPNGYLVV	Mouse
Homo sapiens	YENEEPACEDADEDEDDYHNPGYLVV	Human
Pan troglodytes	YENEEPACEDADEDEDDYHNPGYLVV	Chimpanzee
Rattus norvegicus	NQEPARKNVDEDEDEDDYPEGYLVV	Rat
Myotis brandtii	YENEEPACEDEDDEEEDYHNEGYLVV	Brandt's bat
Mesocricetus auratus	NQEPACESVDEDEDEDDYPNEGYLEV	Golden hamster
Sus scrofa	PPEPVCEDADEDEDEEDYHNEGYLVV	Wild swine
Felis catus	VTSEPACEDDDEDEEEDYPNEGYLEV	Cat
Panthera tigris	ENEEPACEDDDEDEEEDYPNEGYLEV	Tiger
Bos taurus	PPEAACEDADEDDYEEDYNNEGYLVV	Cow

B)

WT-LAT: Asp-Ala-Asp-Glu-Asp-Glu-Asp-Asp-Tyr[132]
LAT-NIL: Gly-Ala-Ser-Gly-Ser-Asn-Gly-Asn-Tyr[132]

C)

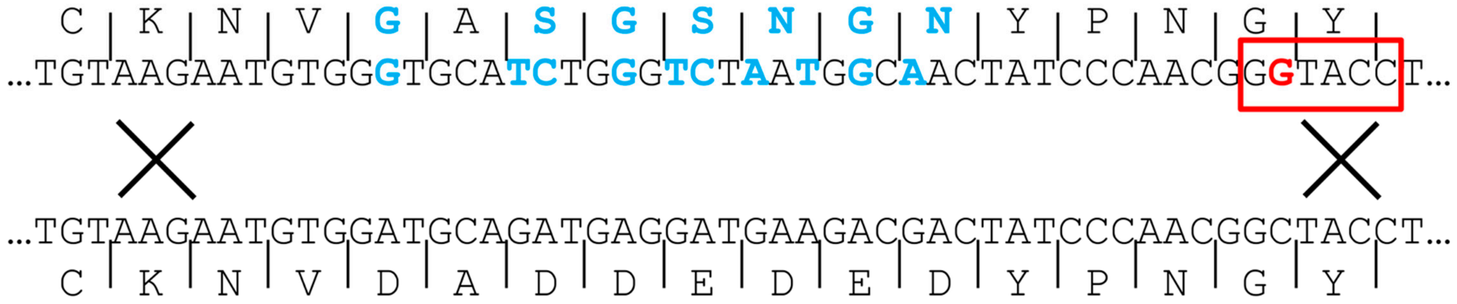

Supplementary Figure S1

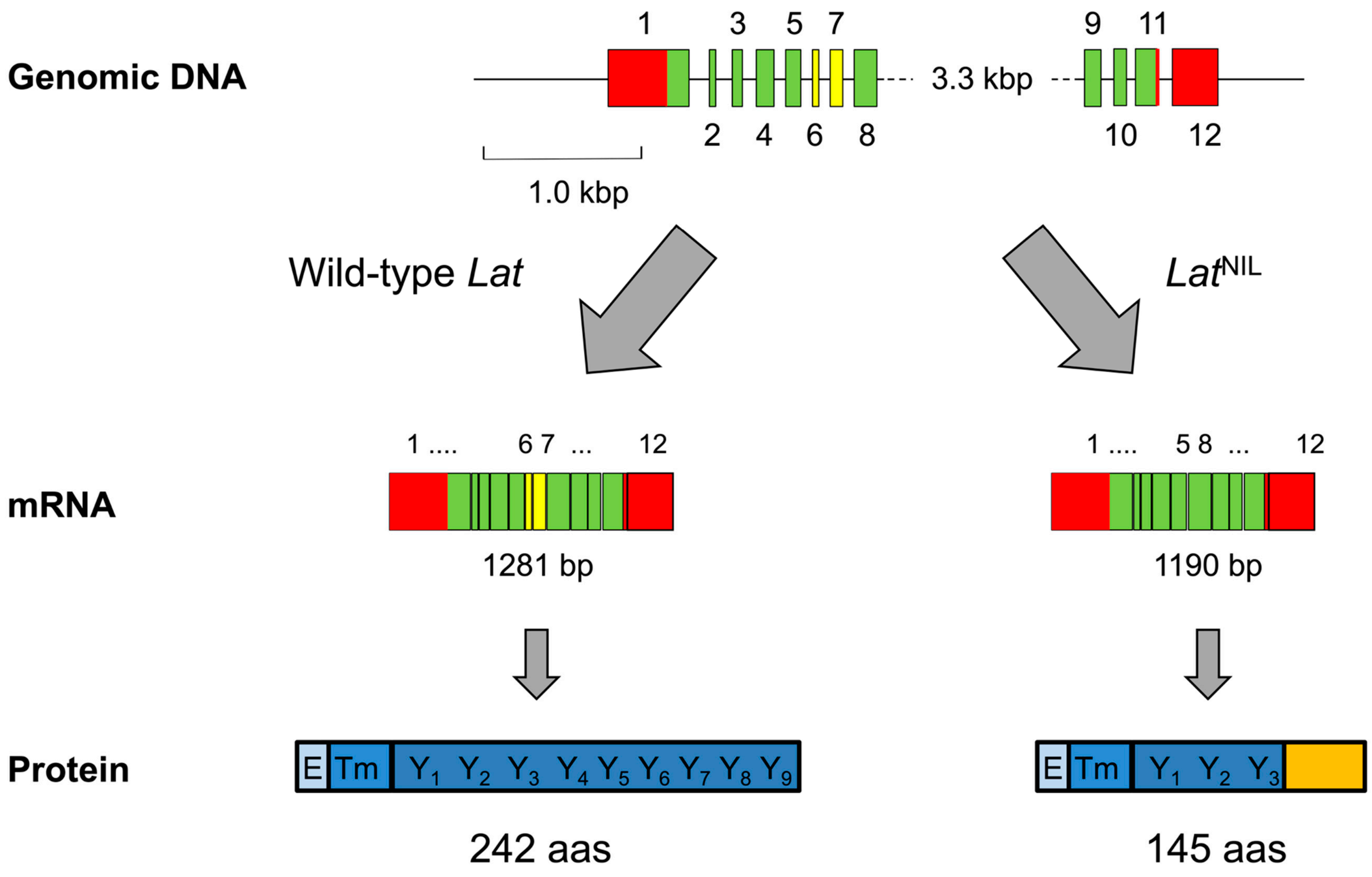

Supplementary Figure S2

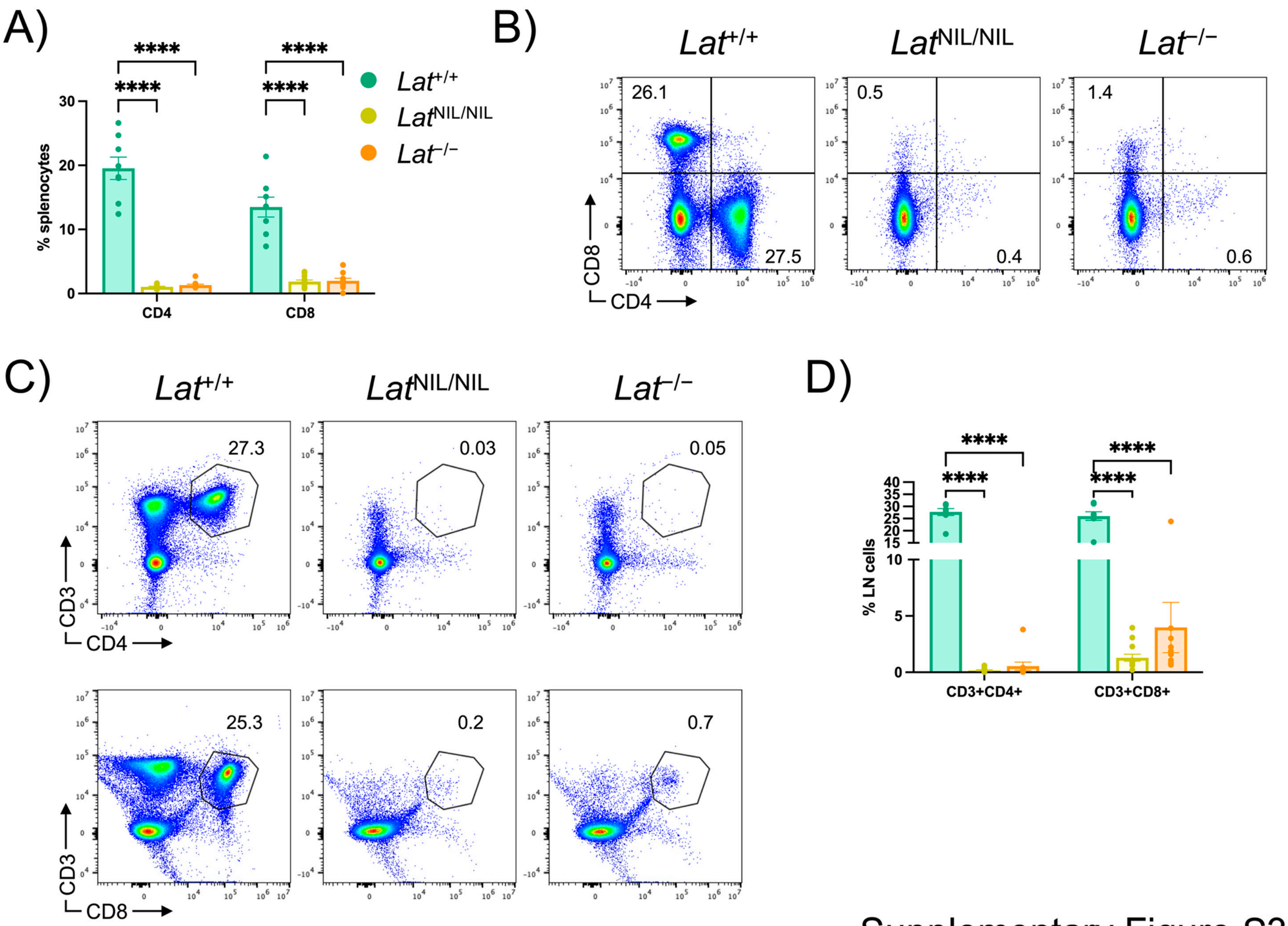

Supplementary Figure S3

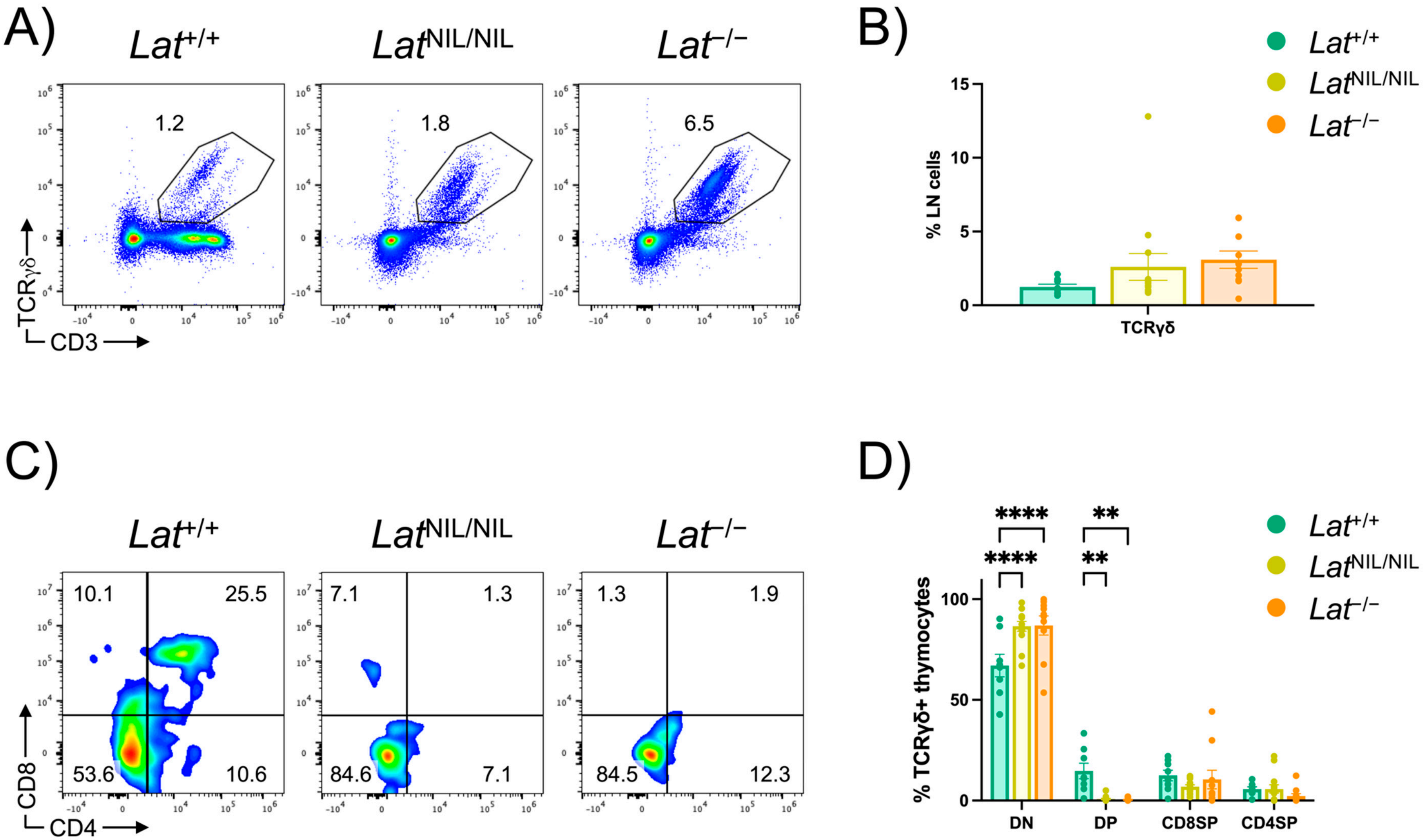

Supplementary Figure S4

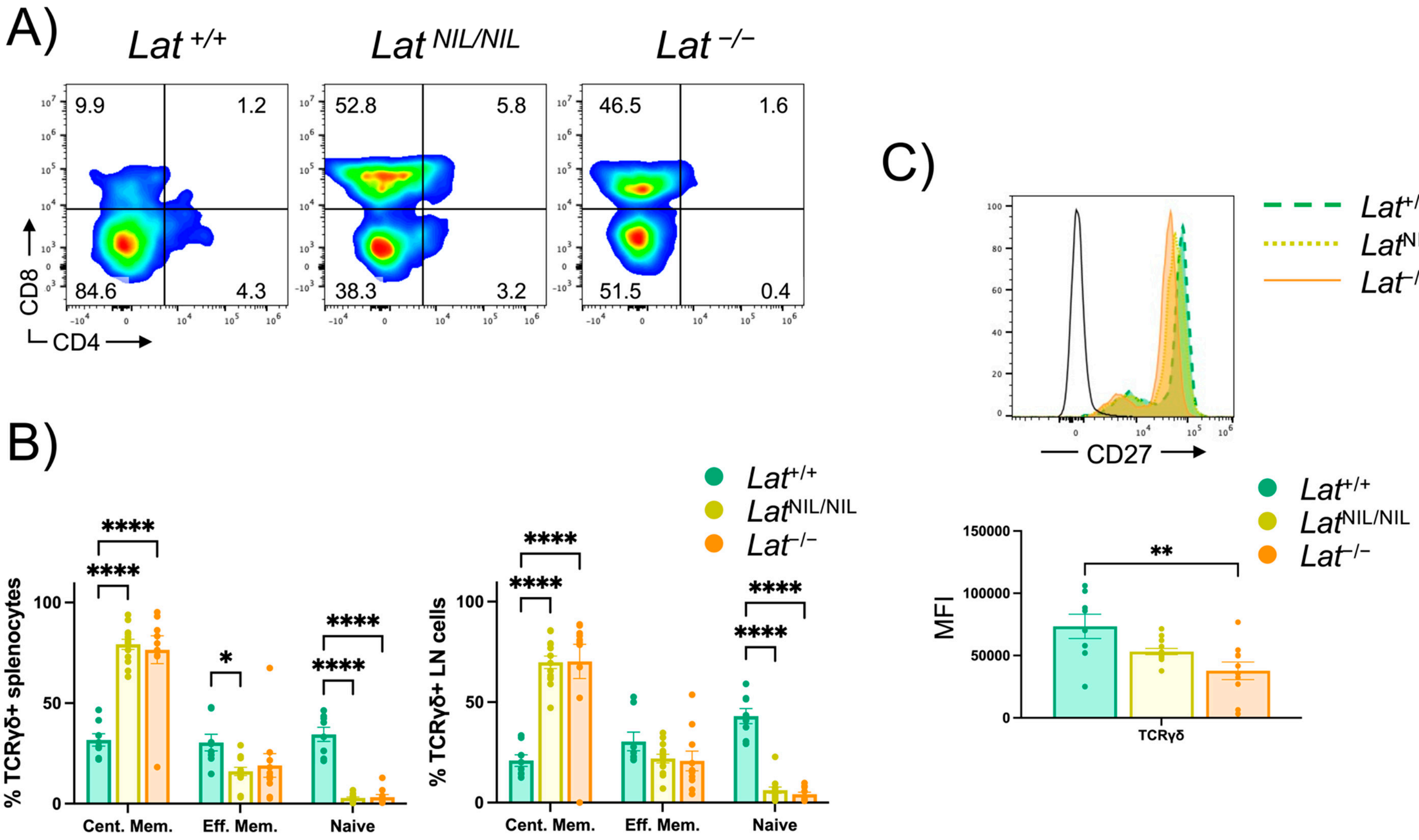

Supplementary Figure S5

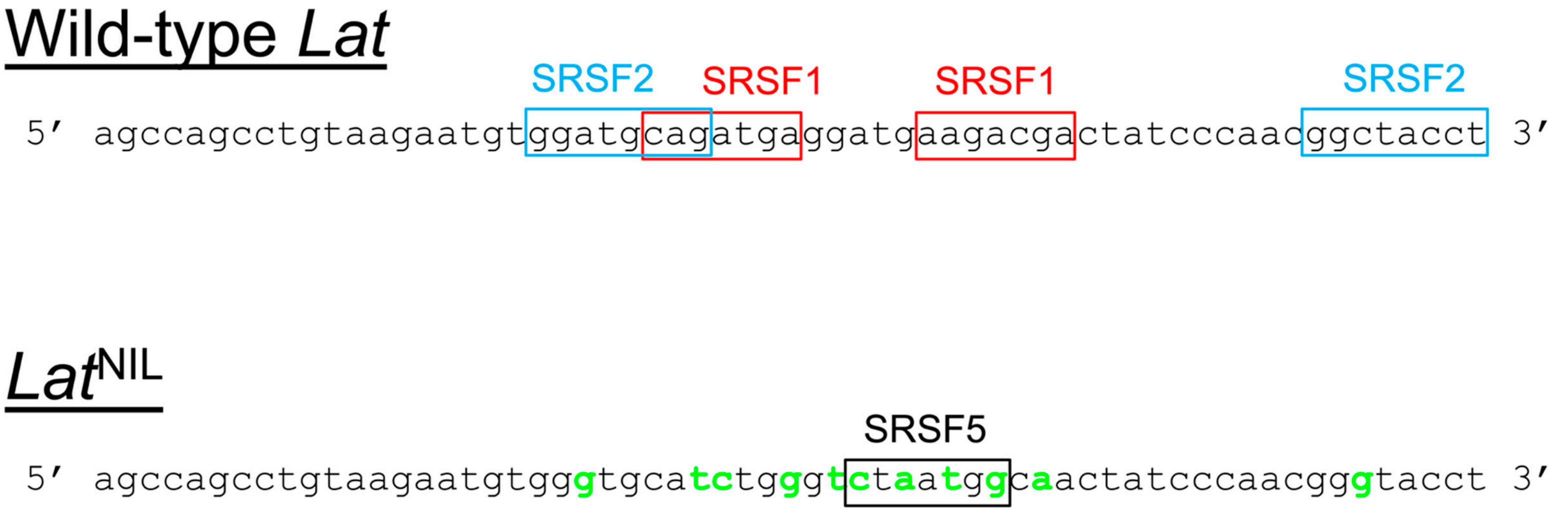

Supplementary Figure S6

Supplementary Figure S1. Amino acid sequence of the negatively charged fragment of LAT in different species and changes to introduce in mouse *Lat*^{NIL} mutant. A) Amino acid sequence preceding the Tyrosine 132 (mouse LAT, marked in yellow) is shown. Marked in green are shown the negatively charged amino acids. B) Amino acid sequence of residues preceding mouse LAT tyrosine 132 (WT-LAT) and sequence of the corresponding to LAT-NIL mutant. C) The top panel shows a segment of the 139 nucleotides ssODN used as a template for mutagenesis (with the amino acids corresponding to each codon in single-letter code), and mutations in the sequence are shown in blue. The silent mutation introducing a KpnI site (red box) is shown in red. The bottom panel shows the wild-type sequence with amino acid translation. The end of exon 7 corresponds to the sequence shown in the bottom panel (TACCT).

Supplementary Figure S2. Schematic representation of the transcription and translation of wild-type LAT and the *Lat*^{NIL} mutant. The top panel shows the exon-intron structure of *Lat* gene at the genomic level. Exons are depicted as boxes, with the untranslated 5' and 3' regions (in exons 1 and 12, respectively) highlighted in red. Coding regions within the exons are shown in green, except exons 6 and 7 (which are deleted in the *Lat*^{NIL} mRNA), shown in yellow. Middle panel illustrates the mRNAs corresponding to wild-type *Lat* or *Lat*^{NIL}. The bottom panel depicts the proteins translated from the corresponding mRNAs. E stands for extracellular and Tm refers to the transmembrane segment. In LAT^{NIL} protein, the new sequence incorporated due to the change in the reading frame is shown in orange.

Supplementary Figure S3. *Lat*^{NIL} mutation depletes CD4^{+} and CD8^{+} T cell populations in the spleen and lymph nodes. A) Bar graphs representing the mean values of CD4^{+} and CD8^{+} cell percentages in the spleens of *Lat*$^{+/+}$ (n = 11), *Lat*$^{+/NIL}$ (n = 4), and *Lat*$^{NIL/NIL}$ (n = 19) mice aged from 10 to 50 weeks. Brackets on each bar represent the standard error mean. B and C) Two-color plots show staining of total lymphocytes obtained from the lymph nodes from the three types of mice. One representative experiment is shown. D) Bar graphs representing the mean values of CD3^{+}CD4^{+} and CD3^{+}CD8^{+} cell percentages in the lymph nodes of *Lat*$^{+/+}$, *Lat*$^{+/NIL}$, and *Lat*$^{NIL/NIL}$ mice. **** indicates $p<0.0001$.

Supplementary Figure S4. Analysis of γδ T cells in *Lat*$^{NIL/NIL}$ and *Lat*$^{-/-}$ mice. A) Analysis of CD3 and TCR γδ expression in lymph node cells from 10 to 50 weeks old wild-type, *Lat*$^{NIL/NIL}$, and *Lat*$^{-/-}$ mutant mice. Percentages of cells are shown in each gate. B) Bar graphs representing the percentages of TCR γδ cells in the lymph nodes from wild-type (*Lat*$^{+/+}$, n = 8), *Lat*$^{NIL/NIL}$ (n = 13), and LAT-knockout (*Lat*$^{-/-}$, n = 10) mice. Brackets on each bar represent the standard error mean. C) CD4 and CD8 expression by TCRγδ^{+} thymocytes. One representative experiment is shown. D) Bar graphs showing the mean percentages of CD4^{-}CD8^{-} double negative (DN), CD4^{+}CD8^{+} double positive, CD8^{+} simple positive (CD8SP), and CD4^{+} simple positive TCRγδ^{+} thymocytes. ** indicates $p<0.01$ and **** indicates $p<0.0001$.

Supplementary Figure S5. Analysis of γδ T cells in lymph nodes from in *Lat*$^{NIL/NIL}$ and *Lat*$^{-/-}$ mice. A) CD4 and CD8 expression analysis in CD3^{+}TCRγδ^{+} LN cells from wild-type, *Lat*$^{NIL/NIL}$, and *Lat*$^{-/-}$ mutant mice. Percentages of cells are shown in each quadrant. One representative experiment is shown. B) Bar graphs representing the mean percentages of Central Memory (CD44^{hi}CD62L^{hi}), Effector Memory (CD44^{hi}CD62L$^{low/neg}$), and Naive (CD44$^{low/neg}$CD62L^{hi}) γδ T cells from the spleen (left panel) or lymph nodes (right panel) from wild-type (*Lat*$^{+/+}$, n = 8), *Lat*$^{NIL/NIL}$ (n = 13), and LAT-knockout (*Lat*$^{-/-}$, n = 10) mice. Brackets on each bar represent the standard error meanr. C) Analysis of CD27 expression in γδ T cells from the lymph nodes of wild-type, *Lat*$^{NIL/NIL}$, and *Lat*$^{-/-}$ mutant mice (upper panel). The bar graph (lower panel) represents the

mean value of CD27 Mean Fluorescence Intensity in lymph node γδ T cells. * indicates $p<0.05$; ** indicates $p<0.01$; **** indicates $p<0.0001$.

Supplementary Figure S6. Potential effects on splicing regulatory elements of the changes introduced in *Lat*$^{\mathbf{NIL}}$. Analysis of exon 7 sequences in wild-type and *Lat*^{NIL} using the ESEfinder tool (https://esefinder.ahc.umn.edu/). Putative binding sites for serine/arginine-rich splicing factors SRSF1 (red boxes), SRSF2 (blue boxes), and SRSF5 (black box) are shown. Mutations in the sequence of the *Lat*^{NIL} allele are shown in green.
